# Supplementary material for: Metabolite profiling and bioactivity of Cicerbita alpina (L.) Wallr. (Asteraceae, Cichorieae)
Source: Plants (Basel). 2023 Feb 23;12(5):1009. doi: 10.3390/plants12051009 (PMC10005263; doi:10.3390/plants12051009)
Supplement: Supplementary file 1 [file plants-12-01009-s001.zip › plants-2219578-supplementary.pdf]

Metabolite Profiling and Bioactivity of *Cicerbita alpina* (L.) Wallr. (Asteraceae, Cichorieae)

Dimitrina Zheleva-Dimitrova [1,*], Alexandra Petrova [2], Gokhan Zengin [3], Kouadio Ibrahime Sinan [3], Vessela Balabanova [1], Olivier Joubert [4], Christian Zidorn [5], Yulian Voynikov [6], Rumyana Simeonova [2] and Reneta Gevrenova [1]

1 Department of Pharmacognosy, Faculty of Pharmacy, Medical University-Sofia, 1000 Sofia, Bulgaria
2 Department of Pharmacology, Pharmacotherapy and Toxicology, Faculty of Pharmacy, Medical University-Sofia, 1000 Sofia, Bulgaria
3 Department of Biology, Faculty of Science, Selcuk University, Campus, 42250 Konya, Turkey
4 Institut Jean Lamour, UMR CNRS 7198, Université de Lorraine, CNRS, IJL, F-54000 Nancy, France
5 Pharmazeutisches Institut, Abteilung Pharmazeutische Biologie, Christian-Albrechts-Universität zu Kiel, 24118 Kiel, Germany
6 Department of Chemistry, Faculty of Pharmacy, Medical University-Sofia, 1000 Sofia, Bulgaria
* Correspondence: dzheleva@pharmfac.mu-sofia.bg

Supplementary material

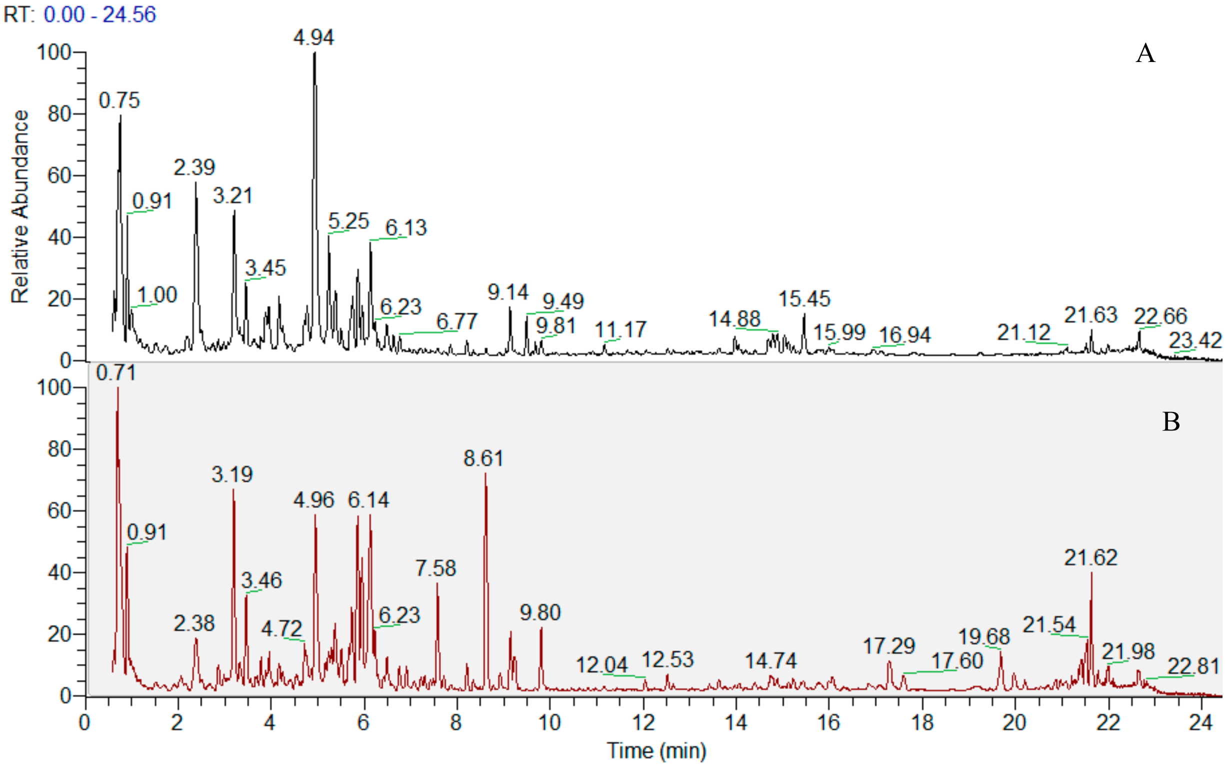

Figure S1. Total ion chromatograms (TIC) of studied *C. alpina* extracts in negative ion mode; A-leaves, B-flowering heads

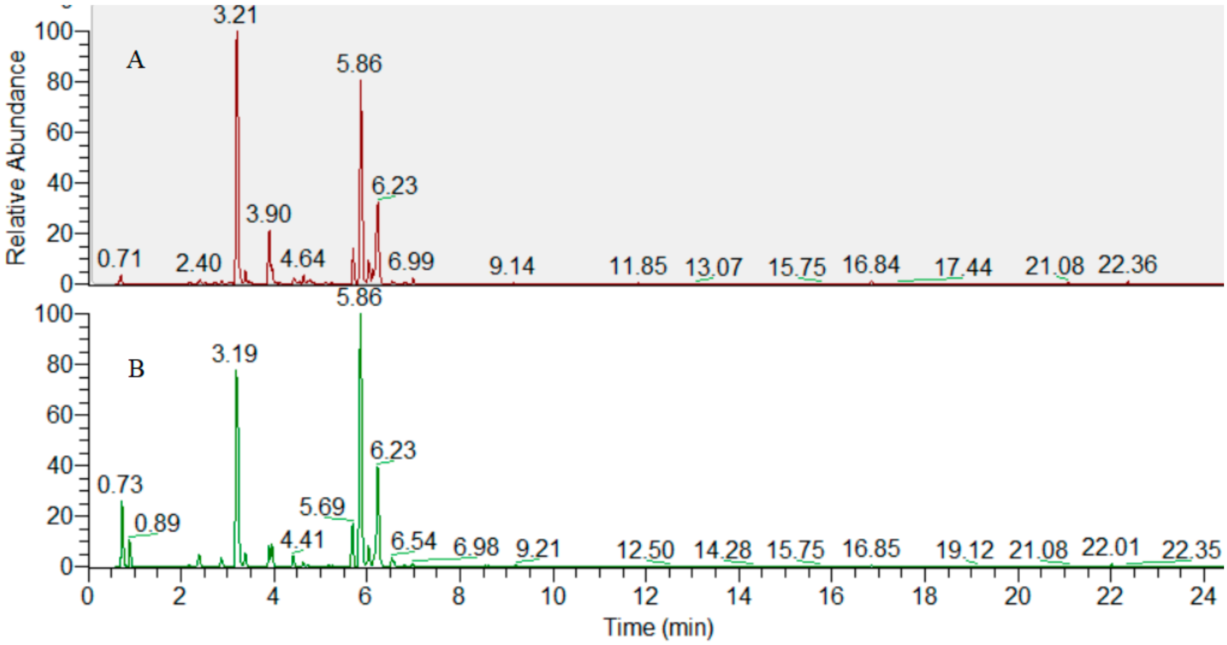

Figure S2. Extracted ion chromatograms of AQAs in *C. alpina* extracts A-leaves, B-flowering heads

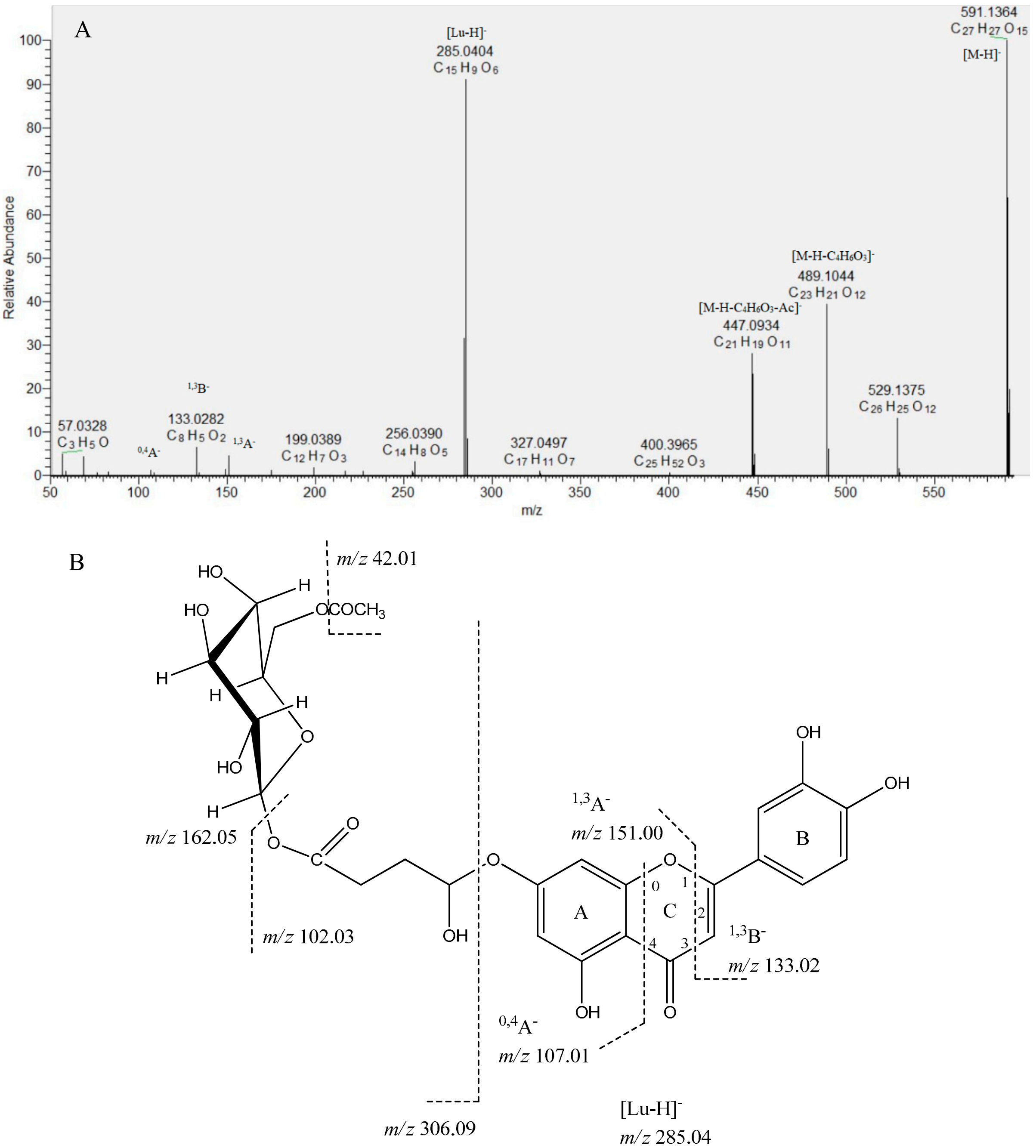

Figure S3. (-) ESI/MS-MS spectrum of luteolin 7-hydroxybutyryl-*O*-acetylhexoside (**83**) (A); Possible fragmentation and preliminary structure of luteolin 7-hydroxybutyryl-*O*-acetylhexoside (83) (B)

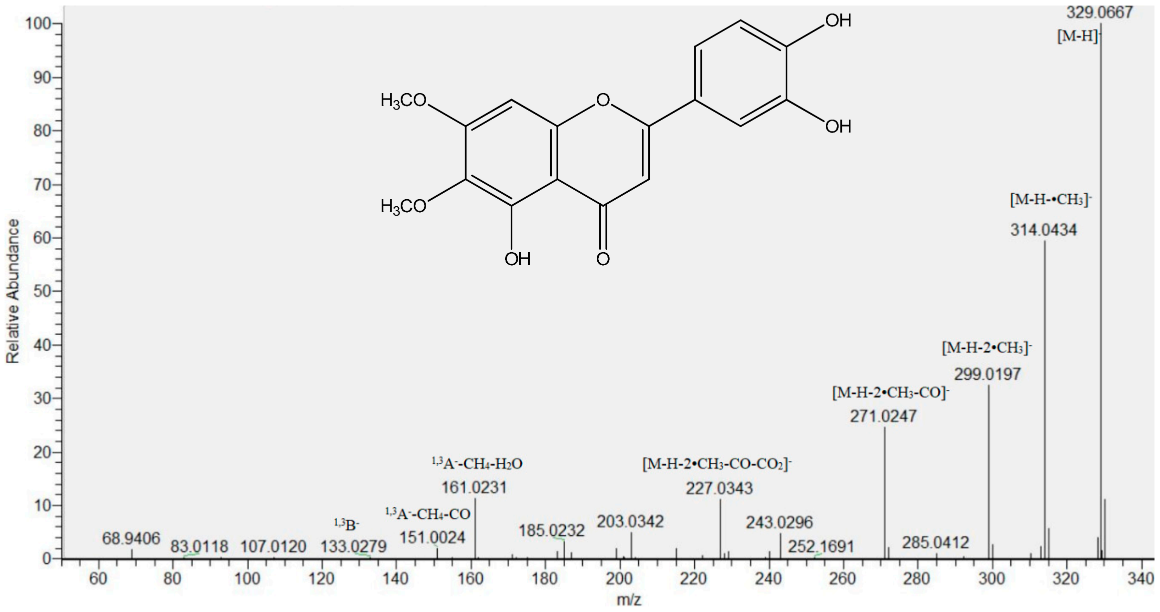

Figure S4. (-) ESI/MS-MS spectrum of cirsiliol (94)

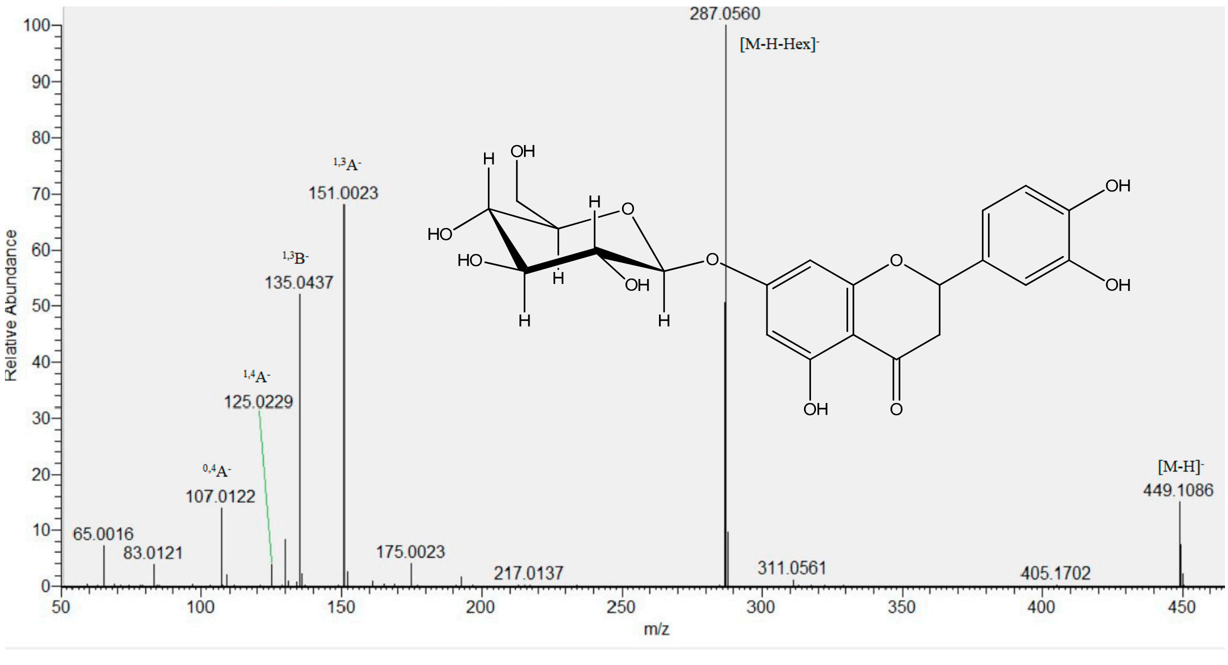

Figure S5. (-) ESI/MS-MS spectrum of eriodyctiol 7-*O*-hexoside (69)

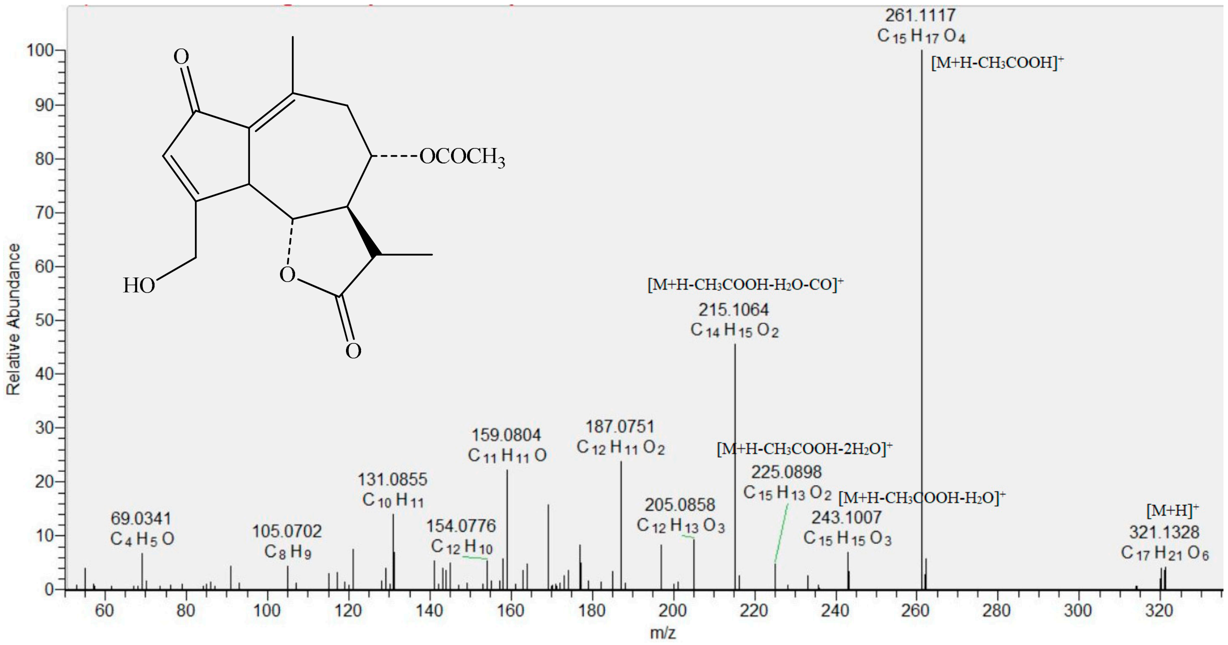

Figure S6. (+) ESI/MS-MS spectrum of 8-acetyl-11β,13-dihydrolactucin (**103**)

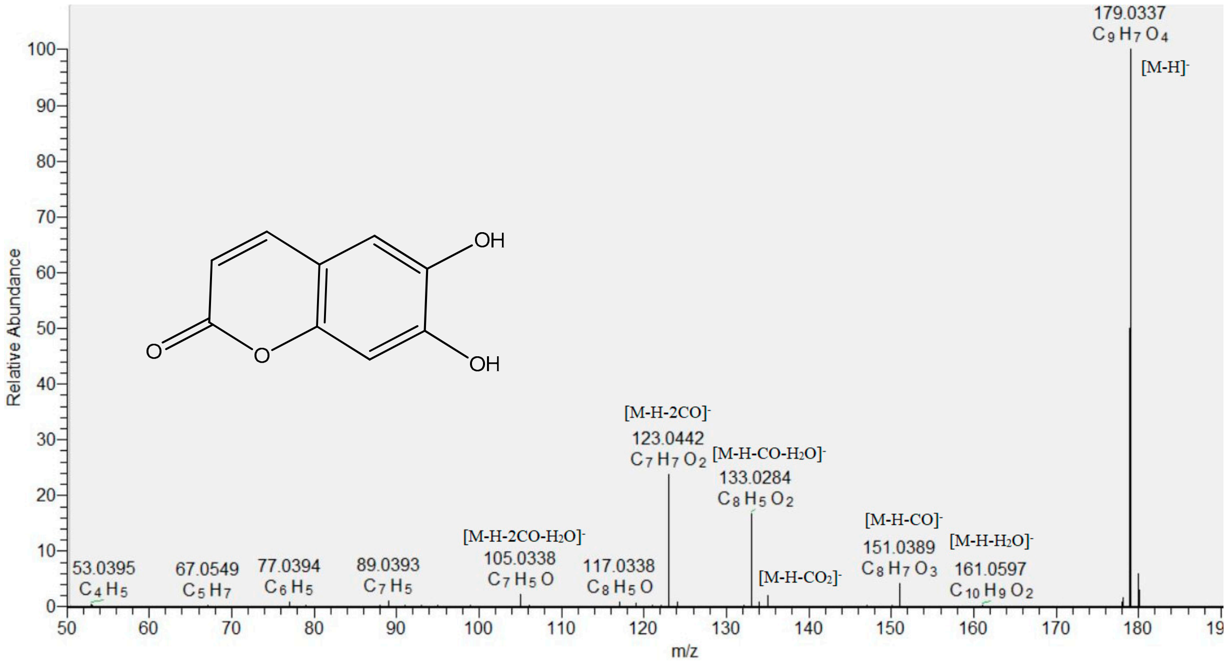

Figure S7. (+) ESI/MS-MS spectrum of aesculetin (**108**)

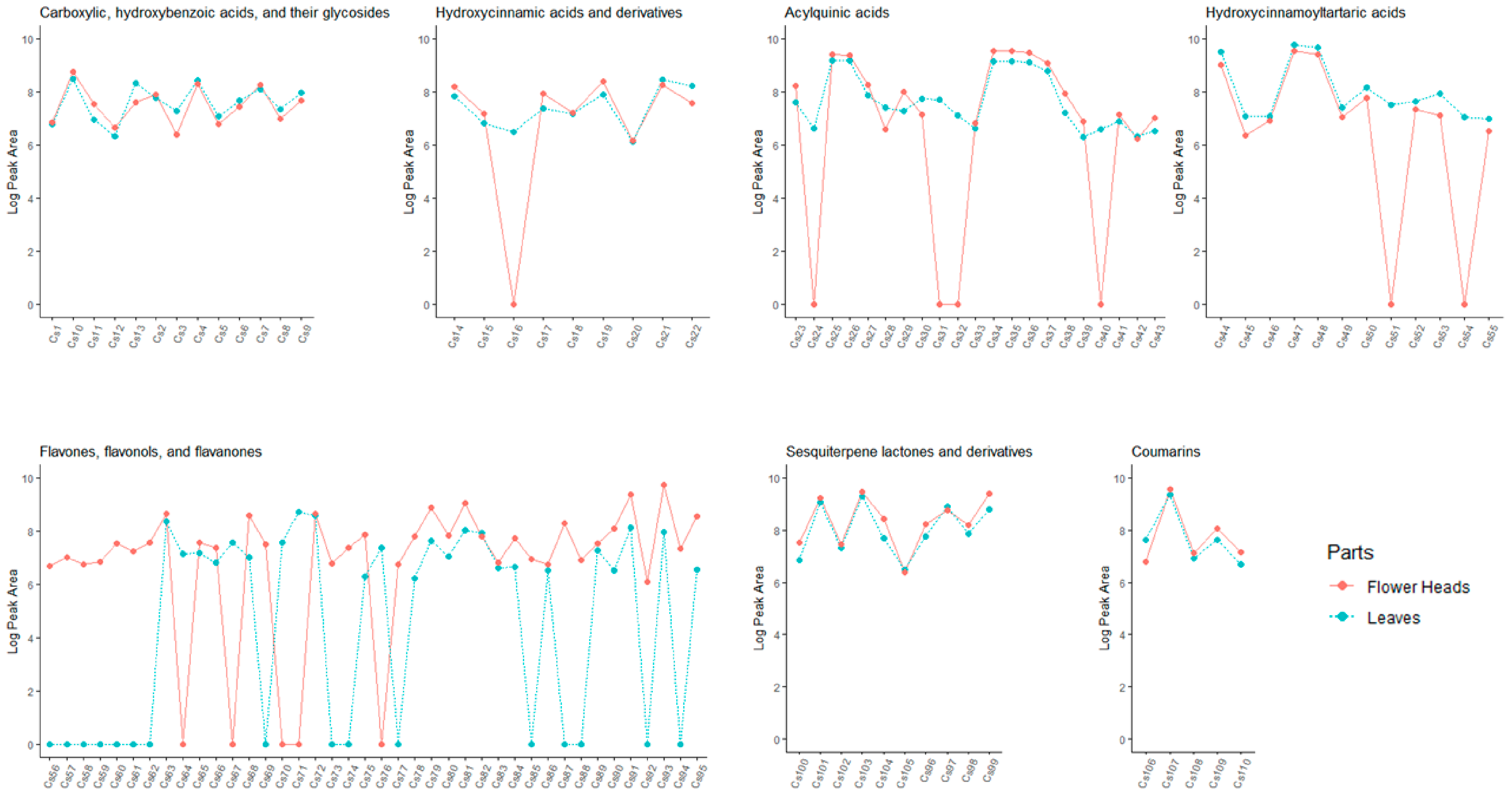

Figure S8. Variation of secondary metabolites among the studied *C. alpine* extracts.
